# Elevated baseline circulating platelet-to-lymphocyte ratio and survival in initial stage Ⅳ gastric cancer patients: A meta-analysis

**DOI:** 10.1371/journal.pone.0265897

**Published:** 2022-04-18

**Authors:** Guoming Hu, Shimin Wang, Songxiang Wang, Liming Huang

**Affiliations:** 1 Department of General Surgery (Breast and Thyroid Surgery), Shaoxing People’s Hospital, Shaoxing Hospital, Zhejiang University School of Medicine, Shaoxing, Zhejiang, China; 2 Department of Nephrology, Shaoxing People’s Hospital, Shaoxing Hospital, Zhejiang University School of Medicine, Shaoxing, Zhejiang, China; Universitá Sapienza di Roma, ITALY

## Abstract

**Background:**

Systemic inflammatory response (SIR) plays important roles in initiation, promotion and progression of tumor. However, the prognostic role of baseline circulating platelet–to–lymphocyte ratio (PLR) (known as a marker of SIR) in human initial stage Ⅳ gastric cancer (GC) remains controversial. Hence, we performed this meta-analysis to assess the value of it in prognosis prediction for these patients.

**Materials and methods:**

We searched PubMed, Embase and EBSCO to identify the studies and computed extracted data with STATA 12.0.

**Results:**

A total of 3025 patients with initial stage Ⅳ GC from 13 published studies were incorporated into this meta-analysis. We found that elevated baseline circulating PLR was significantly associated with decreased overall survival (OS), but not with progression–free survival (PFS) in stage Ⅳ GC patients. However, in stratified analyses, high PLR was only associated with worse 1-year and 2-year OS, but not with 3-year or 4-year OS; In addition, it was considerably related with reduced 6-month PFS, but not with 1-year or 2-year PFS. Moreover, high PLR markedly correlated with peritoneal metastasis of GC.

**Conclusion:**

Elevated baseline circulating PLR decreased 1-year OS and 6-month PFS in initial stage Ⅳ GC patients, implicating that it is a valuable prognostic index for these patients and modifying the inflammatory responses may have a potential for effective treatment.

## Introduction

Human gastric cancer (GC) is one of the most common fatal malignancies worldwide. Although progress in early diagnosis and therapeutic strategies have benefited these patients, the advance in prognosis prediction especially in initial stage Ⅳ GC still remains poor. Recently, several systemic inflammatory response (SIR)—related hematological factors have been extensively investigated to risk-stratify cancer patients to improve treatment selection and to predict survival in many types of cancers including stage Ⅳ GC.

The platelet–to–lymphocyte ratio (PLR), known as a marker of the SIR, which can easily be measured on the basis of absolute platelets and lymphocytes in the clinical setting, has been regarded as a potential prognostic index in various cancers [1]. Previous studies have reported that circulating PLR was remarkably associated with clinical outcomes of GC patients [2, 3]. However, different stages indicate very differential survival of cancer; and SIR will also vary as tumor progresses. They haven’t clarified the association between such index and prognosis in initial stage Ⅳ GC patients in those studies. Although many researchers have investigated the value of baseline circulating PLR in prognosis prediction for stage Ⅳ GC patients, their results were not consistent even controversial [4–6]. A re-assessment is therefore warranted. Moreover, the potential of PLR in peripheral blood before treatment as an effective prognostic index and therapeutic strategy is necessary to be explored.

In this study, we performed the meta-analysis to quantitatively summarize the association between baseline circulating PLR and clinical outcomes such as overall survival (OS) and progression–free survival (PFS) in initial stage Ⅳ GC patients, and thereby provided more evidence on the clinical value of PLR as a prognostic index for these patients.

## Methods

### Search strategy

We searched PubMed, Embase and EBSCO for studies assessing the PLR in peripheral blood before treatment and survival in initial stage Ⅳ GC patients from 1996 to March 31th 2021. The keywords adopted for search were (“platelet to lymphocyte ratio” OR “PLR” OR “inflammation”) AND (“gastric cancer” OR “stomach cancer”) AND (“prognosis” OR “survival”). A total of 524, 836 and 1748 entries were identified in PubMed, Embase and EBSCO respectively.

### Inclusion and exclusion criteria

Inclusion criteria of the meta-analysis were: studies must have (1) been published as original articles in English; (2) assessed human subjects with histopathologically diagnosed with stage Ⅳ GC; (3) provided hazard ratios (HRs) with 95% confidence interval (CI), or Kaplan–Meier curves of high and low circulating PLR before treatment with OS or PFS.

The exclusion criteria were that studies have not been published as research articles or full texts including commentary, case report and letters to editors and conference abstracts; Studies without sufficient data for hazard ratios (HRs) evaluation; studies that detected PLR not in peripheral blood or after treatment or not in stage Ⅳ disease.

### Endpoints

In this meta-analysis, we recorded OS as the primary endpoint; while PFS were regarded as the second endpoint. Individual studies defined cut-offs of PLR and classified patients into high- and low- groups.

### OS and PFS definition

OS was defined as the time from the date of the first curative operation to the date of the last follow-up, or death from any cause; while PFS was the time from the date of the diagnosis until progression or death from any cause.

### Data extraction

Two authors (GM.H. and SX.W.) independently reviewed and extracted information including first author’s name, publication year, number of patients, median age, time of follow-up and cut-off value to determine high PLR. OS, PFS and clinicopathological data including tumor differentiation, peritoneal, liver or lung metastasis etc were extracted from the text or tables.

### Quality assessment

Two independent authors adopted Newcastle–Ottawa Scale (NOS) [7] to assess the quality of individual study, and achieved consensus for each item under the help of the third or more authors. Six or above that the study scored was regarded as high quality.

### Statistical analysis

Relevant data were combined into hazard ratios (HRs) for OS, PFS, and odds ratios (ORs) for clinicopathological features such as tumor differentiation, peritoneal metastasis etc with STATA 12.0 respectively based on the random-effect model if statistical heterogeneity was considerable [8], otherwise, the fixed–effect model was applied [9]. We also adopted sensitivity analysis, Begg’s funnel plot and Egger’s test [10] to determine the influence of individual study on the overall result and potential publication bias respectively. All *P* values were two-sided and below 0.05 was treated as statistical significance.

## Results

### Search results and description of studies

**Fig 1:**
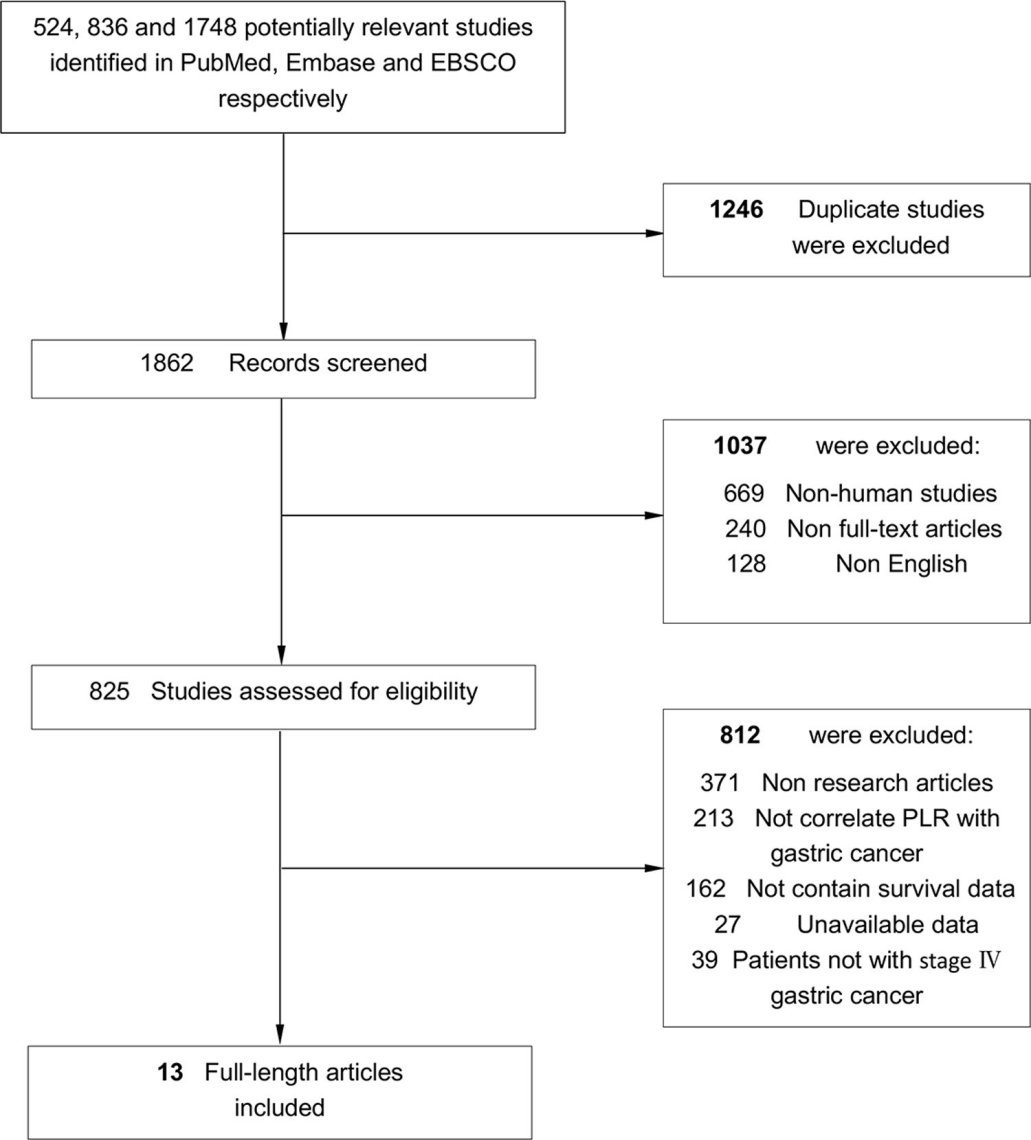
Flow chart diagram of study selection.

Flow chart diagram of study selection was exhibited in Fig 1. Thirteen studies with 3025 patients were ultimately included in this meta-analysis [4–6, 11–20]. And all these studies were scored 6 or above after careful assessment with the Newcastle–Ottawa Scale (NOS). Three studies included patients who underwent non-curative surgery with chemotherapy; One study included patients receiving chemotherapy or not, while the others included patients treated with chemotherapy alone. Four studies evaluated both OS and PFS outcomes; and eight studies evaluated only OS, whereas one study evaluated only PFS. PLR was calculated from pretreatment laboratory data in all these included studies. Characteristics of researches being appropriate for data integration were exhibited in Table 1 and S1 Table in S1 File.

**Table 1:** 

Research	Year	Patients’ No.	M / F	median age (range) (year)	Cut-off value	PLR: (H/L)	Treatment	median follow-up (months)	Clinical outcome	Quality Score (NOS)
Zhao, G.H. et al. [4]	2020	110	84/26	≥65:44.55%; <65:55.45%	≥ 143.39	71/39	chemotherapy	11.6	OS	7
Zhou, D.Y. et al. [14]	2020	537	216/321	55.0 (25, 83)	≥ 284	NR	chemotherapy	NR	OS, PFS	7
Wang, H. et al. [6]	2020	466	327/139	≥60:47.85%; <60:52.15%	≥ 174.79	233/233	chemotherapy	NR	OS, PFS	7
Petrillo, A. et al. [11]	2018	151	97/54	≥62:51.6%; <62:48.4%	≥ 157	76/75	non-curative surgery and chemotherapy	29 (20.4, 37.5)	OS, PFS	8
Huang, Z.H. et al. [16]	2018	136	82/54	55 (28, 85)	≥ 223	47/89	chemotherapy	NR	PFS	6
Wang, J. et al. [12]	2018	273	186/87	56.68±10.73	≥ 201.6	67/206	chemotherapy	NR	OS	7
Aldemir, M.N. et al. [18]	2015	50	30/20	65 (40, 82)	≥ 170	23/27	chemotherapy	NR	OS	7
Wang, F. et al. [17]	2015	120	75/45	68 (32, 82)	≥ 235	60/60	chemotherapy	≤40	OS, PFS	8
Wang, Q. et al. [5]	2014	365	270/95	≥50:76.7%; <50:23.3%	≥ 160	197/168	chemotherapy	NR	OS	7
Lee, S. et al. [19]	2013	174	114/60	(24, 74)	≥ 160	86/88	chemotherapy	14.9 (1.0, 47.9)	OS	7
Mimatsu, K. et al. [15]	2017	33	25/8	(62.0, 79.8)	≥ 200	16/17	non-curative surgery and chemotherapy	NR	OS	7
Zhai, Z. et al. [13]	2021	306	245/61	58 (28, 85)	≥ 128	NR	non-curative surgery and chemotherapy	11	OS	7
Huang, C. et al. [20]	2020	304	198/106	60 (51, 67)	≥ 107.7	263/41	Chemotherapy or not	NR	OS	7

PLR, platelet–to–lymphocyte ratio; M, male; F, female; OS, overall survival; PFS, progression–free survival; NR: not reported.

### Meta-analyses

#### Overall survival (OS)

**Fig 2:**
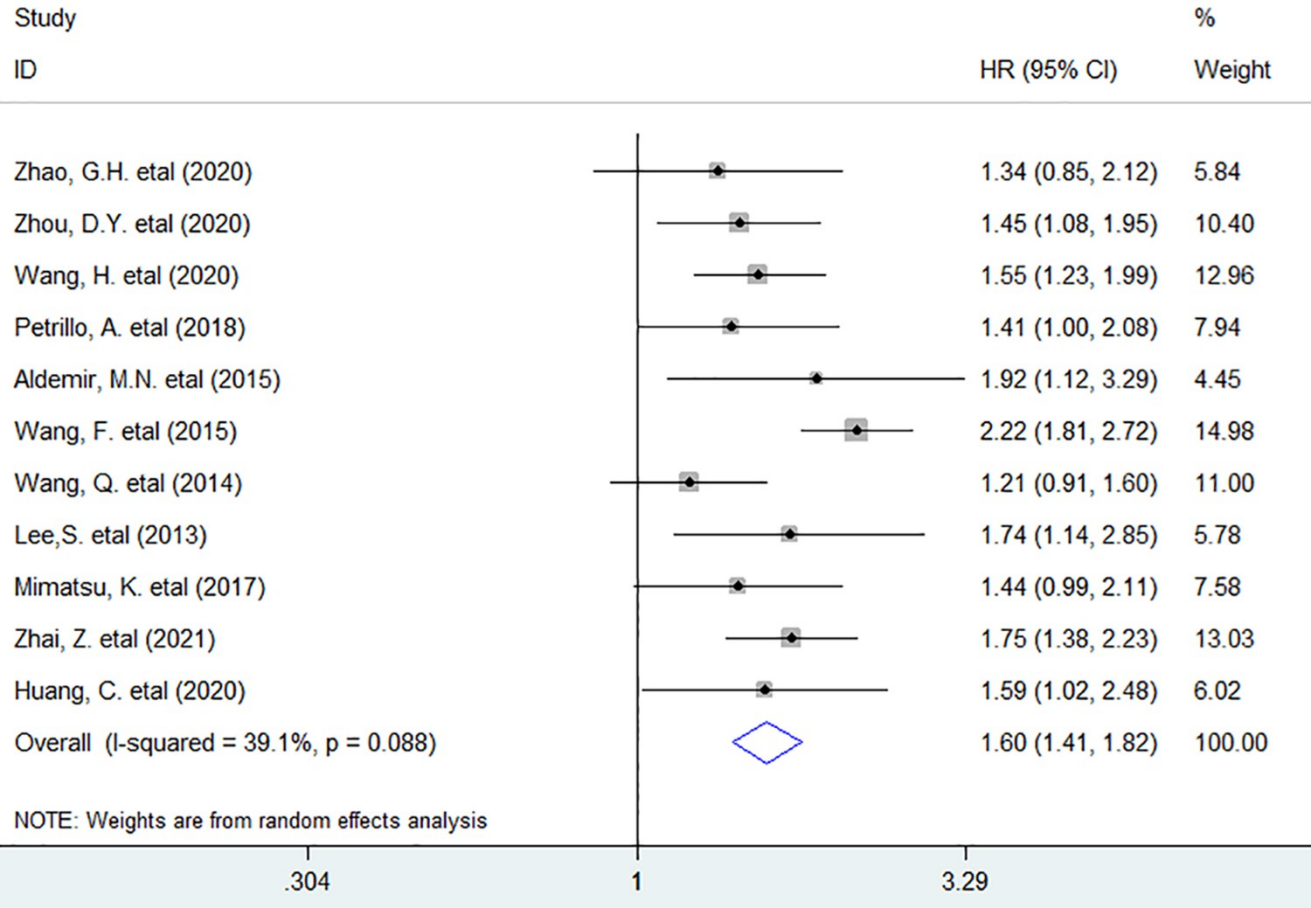
Forest plots describing HR of the association between elevated baseline circulating PLR and OS in initial stage Ⅳ GC patients. HR: hazard ratio; GC: gastric cancer; OS: overall survival.

Eleven studies involving 2616 patients investigated the association between PLR and OS, and the meta-analysis exhibited that increased baseline PLR in peripheral blood notably decreased OS (HR = 1.60, 95% CI 1.41 to 1.82, *P <* 0.001) in patients with stage Ⅳ GC (Fig 2).

**Fig 3:**
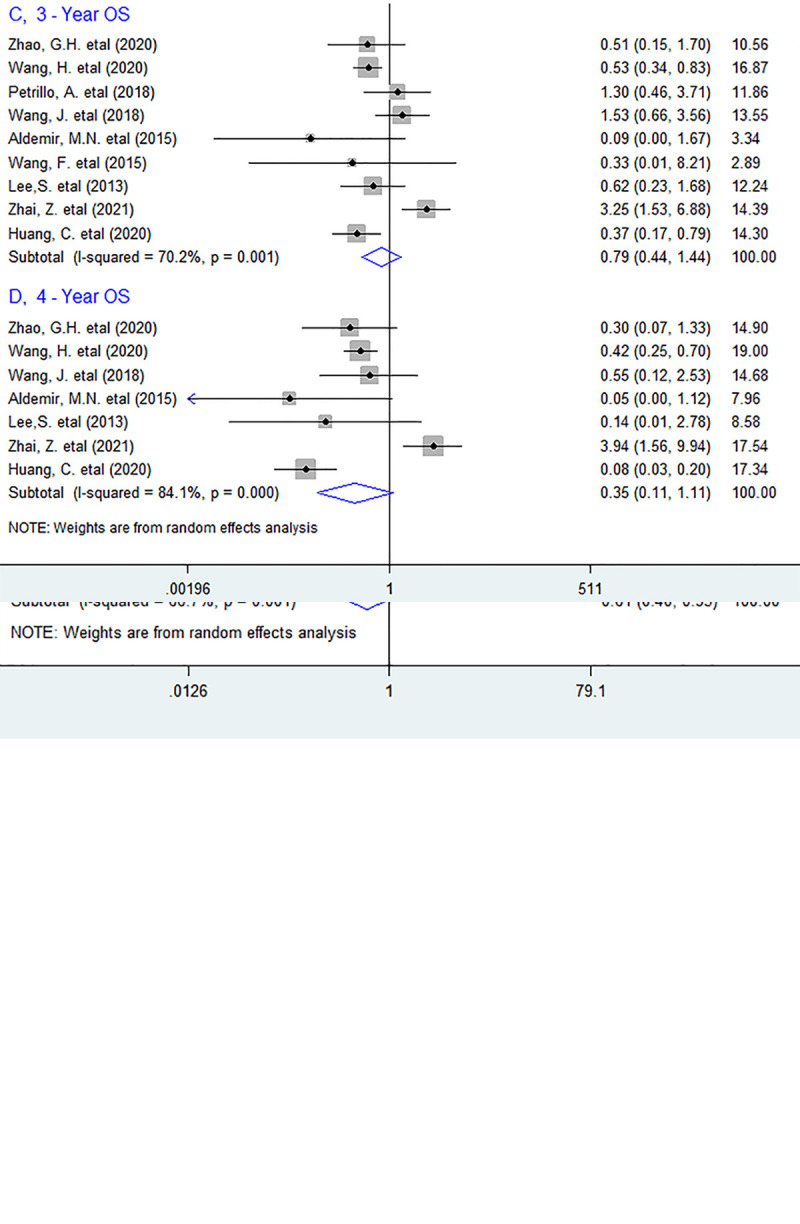
Forest plots describing ORs of the association between elevated baseline circulating PLR and OS at 1-year, 2-year, 3-year and 4-year in initial stage Ⅳ GC patients. OR, odds ratios; GC gastric cancer OS: overall survival.

However, in stratified analyses, we noted that elevated circulating PLR before treatment was only associated with worse 1-year (OR = 0.48, 95% CI 0.36 to 0.64, *P <* 0.001) and 2-year (OR = 0.61, 95% CI 0.40 to 0.95, *P* = 0.028) survival, but not with 3-year (OR = 0.79, 95% CI 0.44 to 1.44, *P* = 0.451) or 4-year (OR = 0.35, 95% CI 0.11 to 1.11, *P* = 0.075) OS (Fig 3).

#### Progression–free survival (PFS)

**Fig 4:**
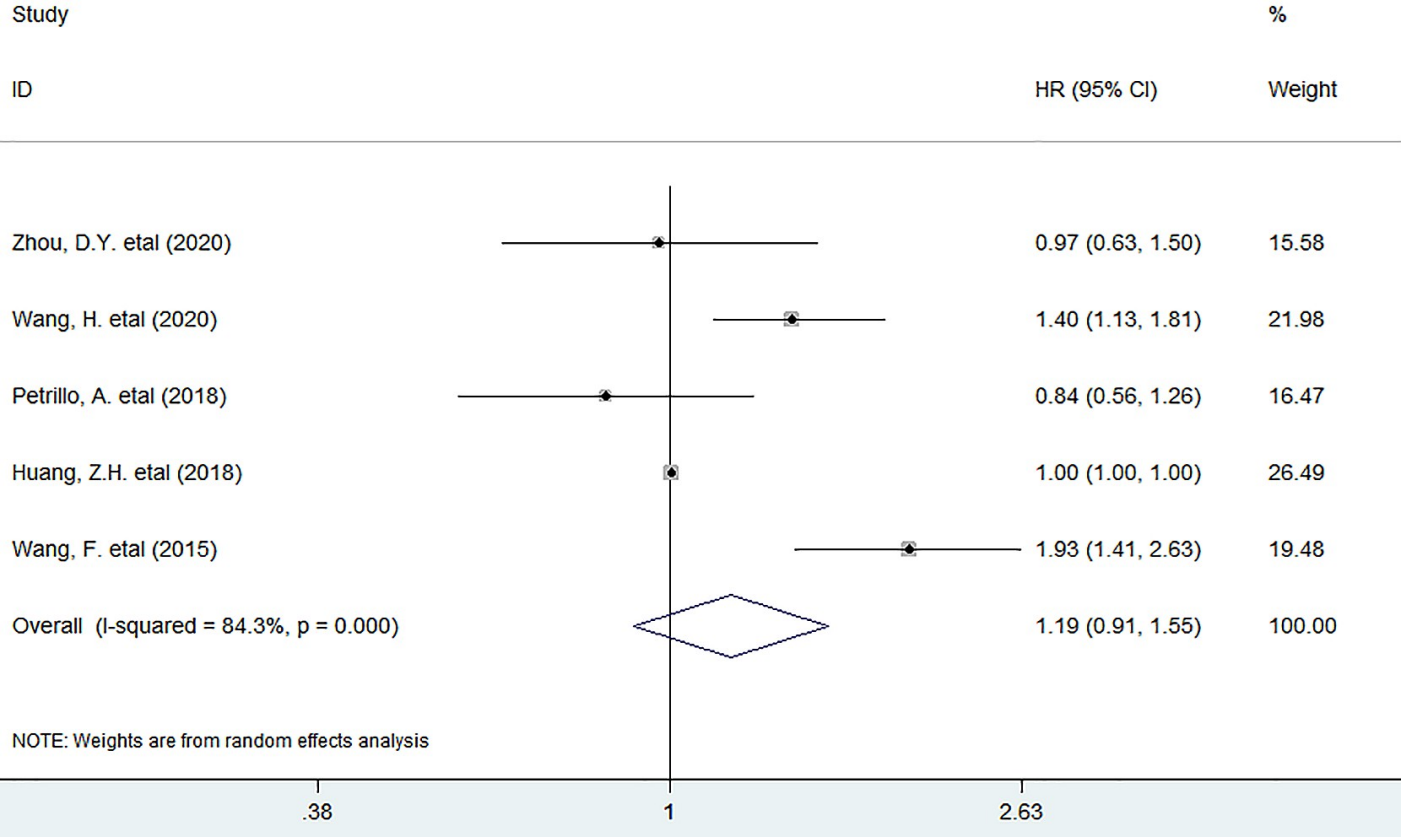
Forest plots describing HRs of the association between elevated baseline circulating PLR and PFS in initial stage Ⅳ GC patients. HR: hazard ratio; GC: gastric cancer; PFS, progression–free survival.

**Fig 5:**
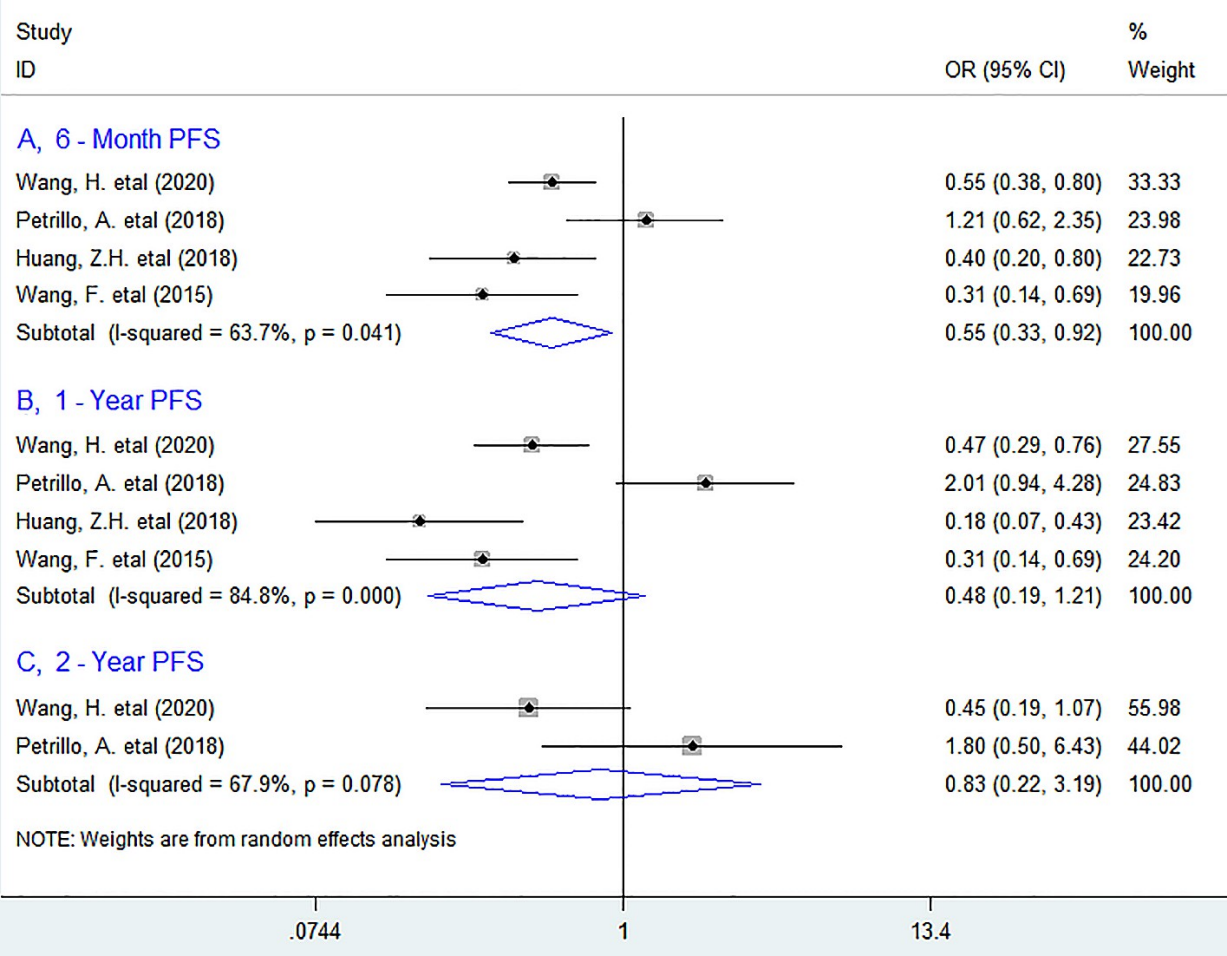
Forest plots describing ORs of the association between elevated baseline circulating PLR and PFS at 6-month, 1-year, 2-year in initial stage Ⅳ GC patients. OR, odds ratios; GC gastric cancer; PFS, progression–free survival.

As for the association between PLR and PFS, five studies have supplied the relevant data. The pooled data indicated that high baseline circulating PLR was not markedly associated with reduced PFS in patients (HR = 1.19, 95% CI 0.91 to 1.55, *P* = 0.213). (Fig 4) in stratified analyses, as shown in Fig 5, the results revealed that it was considerably related with worse 6-month PFS (OR = 0.55, 95% CI 0.33 to 0.92, *P* = 0.022), but not with 1-year (OR = 0.48, 95% CI 0.19 to 1.21, *P* = 0.121) or 2-year PFS (OR = 0.83, 95% CI 0.22 to 3.19, *P* = 0.787).

#### Clinicopathological features

We next investigated whether high baseline circulating PLR correlated with clinicopathological features, and discovered that it was considerably associated with peritoneal metastasis (OR = 1.30, 95% CI 1.04 to 1.63, *P* = 0.024), but not with liver (OR = 1.41, 95% CI 0.84 to 2.39, *P* = 0.195) or lung (OR = 1.03, 95% CI 0.35 to 3.04, *P* = 0.954) metastasis in stage Ⅳ disease (S1A–S1C Fig in S1 File). And there was no correlation between PLR and tumor differentiation (OR = 1.10, 95% CI 0.82 to 1.47, *P* = 0.528) of patients (S1D Fig in S1 File).

### Sensitivity analysis

Sensitivity analysis demonstrated that each included research had no impact on the overall result for OS or PFS (S2 Fig in S1 File).

### Publication bias

Funnel plot and Egger’s test indicated that no significant publication bias existed between combined therapy and OS (*P* = 0.239) or PFS (*P* = 0.715) in patients (S3 Fig in S1 File).

## Discussion

Systemic inflammatory response is closely related to the initiation, promotion and progression of cancer [21]. In this study, we found that high baseline circulating PLR remarkably decreased OS in initial stage Ⅳ GC patients; However, we noted that it didn’t reduce PFS in these patients. In stratified analyses, high PLR was only associated with worse 1-year and 2-year OS, and 6-month PFS, but not with 3-year or 4-year OS, or 1-year, 2-year PFS. In addition, high PLR was considerably correlated with peritoneal metastasis of GC. These findings suggested that high baseline circulating PLR played a critical role in promoting tumor progression and metastasis of GC.

Several potential mechanisms might be responsible for the close association between increased PLR and worse prognosis. Previous researches have demonstrated that platelets play a key role in tumor progression and are associated with poor survival in patients with various types of malignancies [22]. Platelets can induce angiogenesis via the secretion of vascular endothelial growth factor (VEGF) and inhibit anti-tumor response mediated by effector T cells [23]. Platelets can also protect the circulating tumor cells (CTCs) from shearing stresses during circulation [24]. Lymphocytes have an important role in cancer immune surveillance and prevent development of malignancy [25]. The decrease in CD4+ T-helper cells may lead to a suboptimal lymphocyte-mediated immune response to cancer cells [26]. Taken together, we observed that an increase in the platelet count or decrease in the lymphocyte count in the peripheral blood correlate with tumor initiation and progression. Hence, the PLR may help to predict prognosis and reflect the degree of tumor progression in stage Ⅳ GC. However, in this study, we noted that pre-treatment PLR correlated with worse 1-year, 2-year OS and 6-month PFS rather than 3-year, 4-year OS, and 1-year, 2-year PFS. The mechanism underlying such results needs further investigation.

Previous studies have demonstrated that Inflammatory cytokines such as IL-17A and IL-6 were closely linked with cancer associated inflammation [27]. Hence, we thought that the addition of agents (for example: IL-17A mAb, IL-6 mAb) against inflammation to the basic treatment including chemotherapy, palliative surgery etc would probably be a promising strategy for patients with initial stage IV gastric cancer.

There is a limitation that should be noted from this meta-analysis, studies with negative results might not be published, which could cause potential publication bias.

In conclusion, elevated circulating PLR before treatment leads to worse 1-year and 2-year OS and 6-month PFS in initial stage Ⅳ GC patients, implicating that it might be a valuable prognostic index and modifying the inflammatory responses may have a potential for effective treatment for these patients.

## Supporting information

S1 Checklist PRISMA 2009 checklist. (DOC)

S1 File (DOC)

## Abbreviations

PLR: platelet–to–lymphocyte ratio
SIR: systemic inflammatory response
GC: gastric cancer
OS: overall survival
PFS: progression–free survival
HR: hazard rations
OR: odds ratios
Cl: confidence interval
NR: not reported
