# Supplementary material for: Elevated baseline circulating platelet-to-lymphocyte ratio and survival in initial stage Ⅳ gastric cancer patients: A meta-analysis
Source: PLoS One. 2022 Apr 18;17(4):e0265897. doi: 10.1371/journal.pone.0265897 (PMC9015147; doi:10.1371/journal.pone.0265897)
Supplement: S1 File — (DOC) [file pone.0265897.s002.doc]

**Supplementary data**

**Elevated Baseline Circulating Platelet-to-lymphocyte Ratio And Survival In** **Initial Stage Ⅳ** **Gastric Cancer Patients: A Meta-analysis**

Guoming Hu ^1^*, Shimin Wang ^2^, Songxiang Wang^1^, Liming Huang ^1^*

^1^ Department of General Surgery (Breast and Thyroid Surgery), Shaoxing People’s Hospital; Shaoxing Hospital, Zhejiang University School of Medicine, Shaoxing, Zhejiang, China.

^2^ Department of Nephrology, Shaoxing People’s Hospital; Shaoxing Hospital, Zhejiang University School of Medicine, Shaoxing, Zhejiang, China.

*** Corresponding author:**

**E-mail:** Guoming Hu: hgmplj@126.com (GMH); Liming Huang: shaoxinghlm@126.com (LMH).


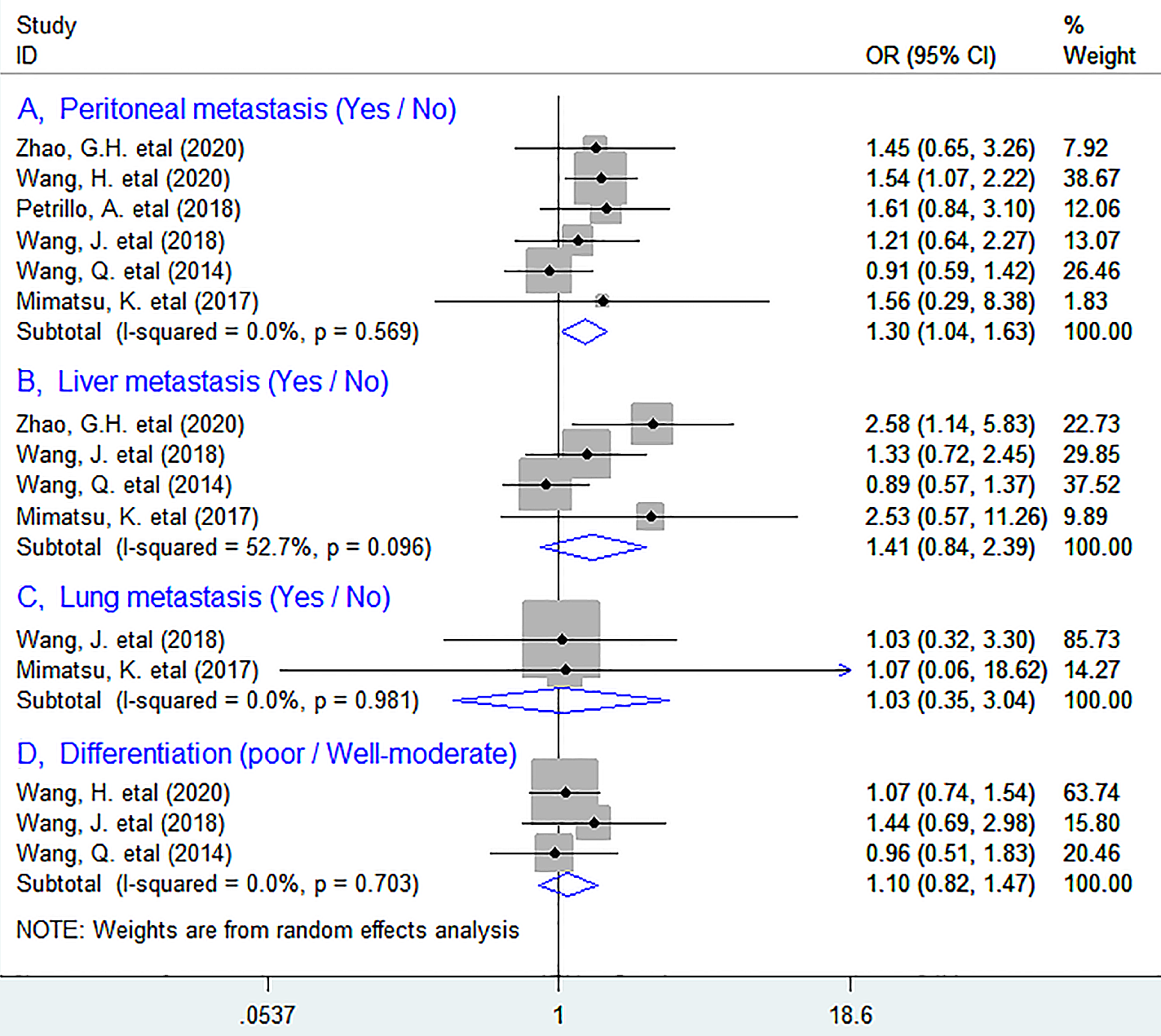


**Fig. S1.** Forest plots indicating ORs of the association between elevated baseline circulating PLR and clinicopathological features including peritoneal (**A**), liver (**B**), lung (**C**) metastasis and tumor differentiation (**D**) in patients. ORs: odds ratios.

**
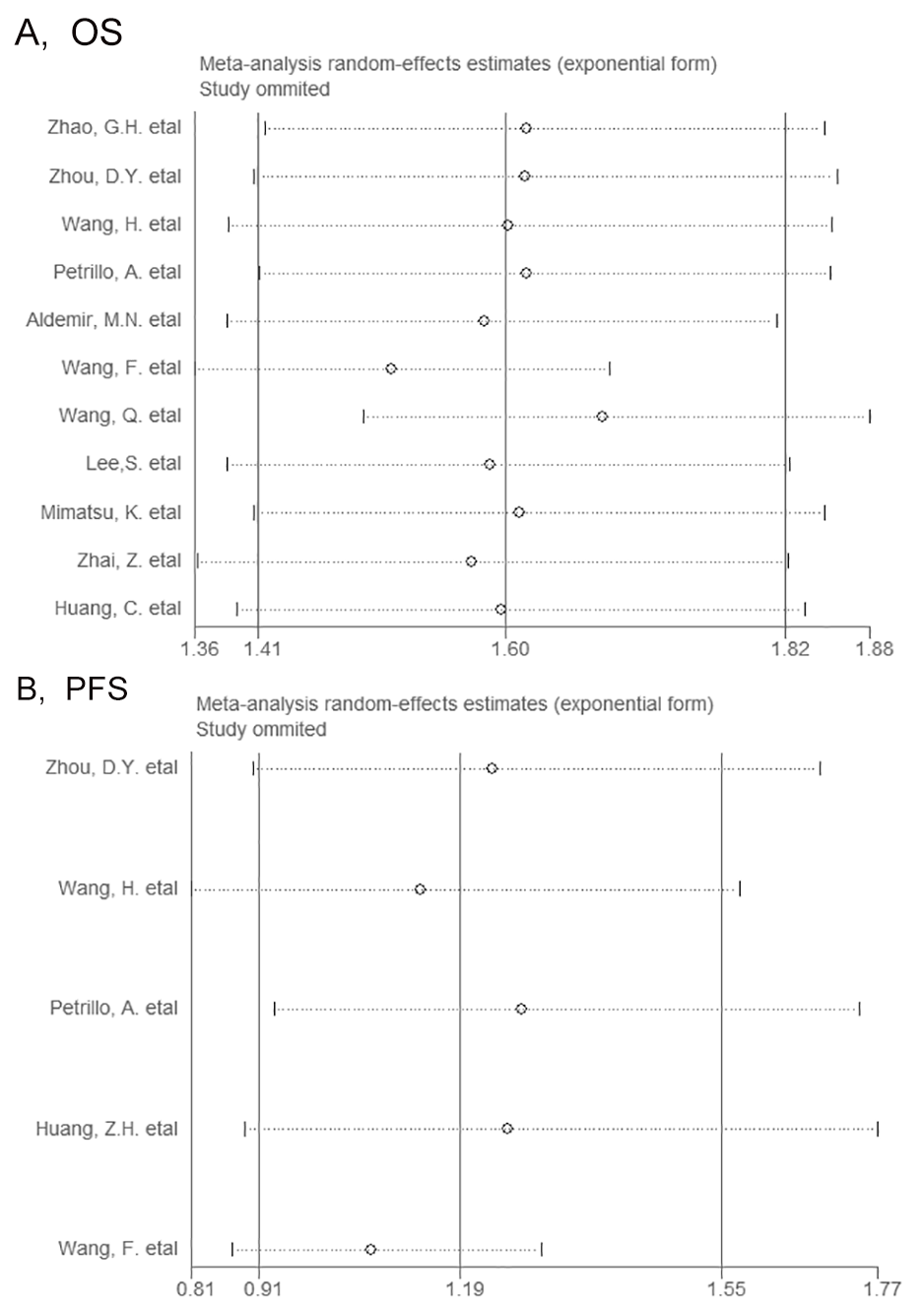
**

**Fig. S2.** Plots describing the influence of individual studies on the overall HRs for OS (**A**) and PFS (**B**) in patients. OS: overall survival; PFS, progression**–**free survival.

**
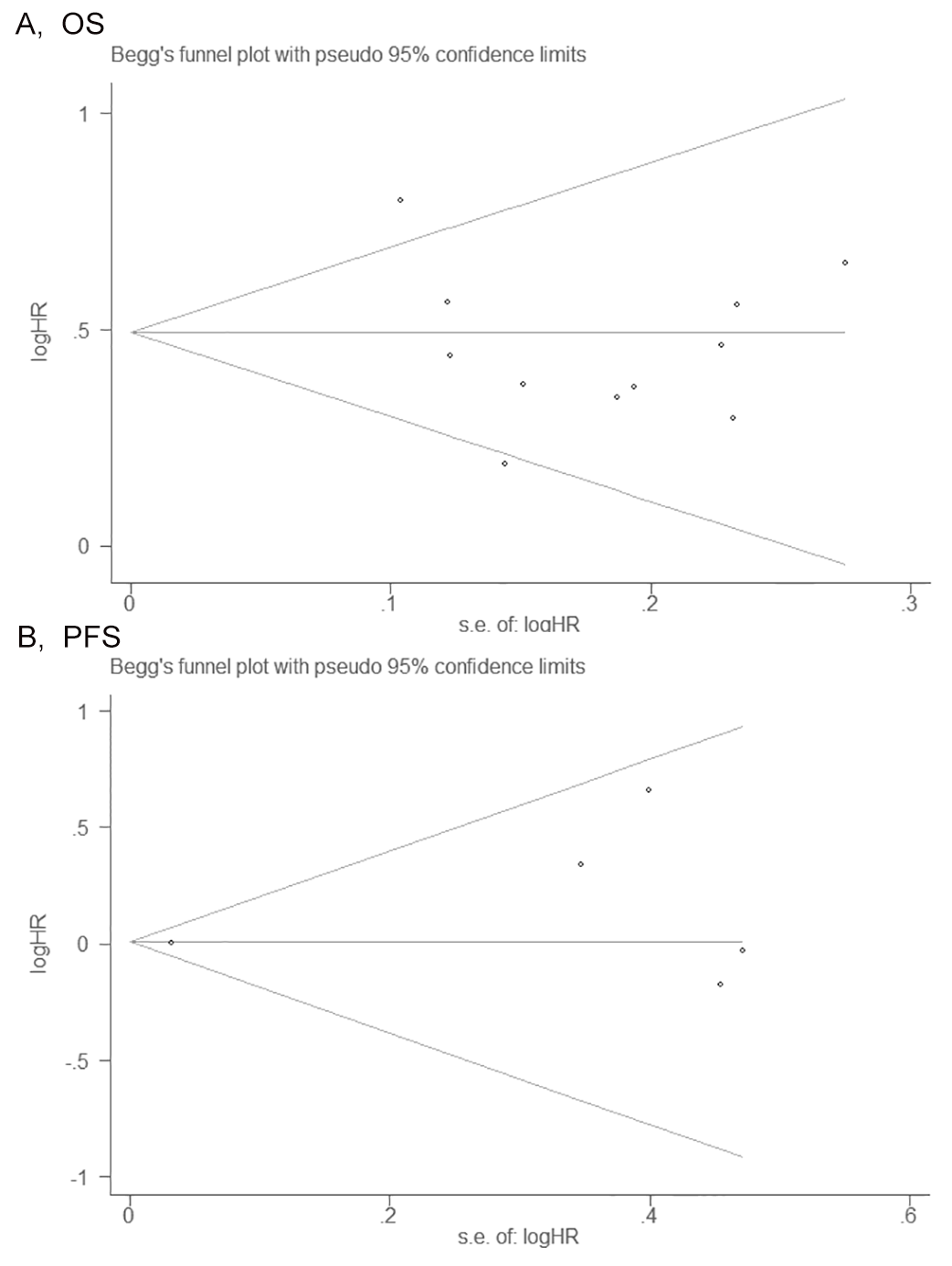
**

**Fig. S3.** Funnel plots displayed the potential publication bias between elevated baseline circulating PLR and OS (**A**) or PFS (**B**) in patients. OS: overall survival; PFS, progression**–**free survival.

**Table S1.** Characteristics of the included studies for OR analysis of clinicopathological features.

| **Research** | **Year** | **No. of Patients** | **PLR: (H/L)** | **Peritoneal metastasis**  **(Yes / No)** | **Liver metastasis (Yes / No)** | **Lung metastasis**  **(Yes / No)** | **Tumor Differentiation**  **(poor/Well-moderate)** |
| --- | --- | --- | --- | --- | --- | --- | --- |
| Zhao, G.H. etal[4] | 2020 | 110 | 71/39 | H:(48/23) L:(23/16) | H:(40/31) L:(13/26) | NR | NR |
| Wang, H. etal[6] | 2020 | 466 | 233/233 | H:(122/111) L:(97/136) | NR | NR | H:(116/117) L:(112/121) |
| Petrillo, A. etal[13] | 2018 | 151 | 76/75 | H:(35/41) L:(26/49) | NR | NR | NR |
| Wang, J. etal[14] | 2018 | 273 | 67/206 | H:(18/49) L:(48/158) | H:(20/47) L:(50/156) | H:(4/63) L:(12/194) | H:(32/13) L:(89/52) |
| Wang, Q. etal[5] | 2014 | 365 | 197/168 | H:(123/65) L:(112/54) | H:(66/123) L:(63/104) | NR | H:(25/115) L:(21/93) |
| Mimatsu, K. etal[18] | 2017 | 91 | 51/40 | H:(4/12)  L:(3/14) | H:(7/9)  L:(4/13) | H:(1/15)  L:(1/16) | NR |

NR: not reported; NA: not applicable; H: high; L: low.
